# Associations Between Laser Treatment Utilization, Regional Access, and Vision-Threatening Diabetic Retinopathy in Northwest Tanzania: A Retrospective Study

**DOI:** 10.7759/cureus.90593

**Published:** 2025-08-20

**Authors:** Richmond Woodward, Christopher Mwanansao, Robert N Peck, Grace Sun

**Affiliations:** 1 Retina Service, Massachusetts Eye and Ear, Boston, USA; 2 Ophthalmology, Bugando Medical Centre, Mwanza, TZA; 3 Internal Medicine, Weill Cornell Medicine, New York, USA; 4 Ophthalmology, Weill Cornell Medicine, New York, USA

**Keywords:** diabetic retinopathy, laser photocoagulation, sub-saharan africa, vision threatening diabetic diabetic retinopathy, vtdr

## Abstract

Background

The influence of proximity to laser treatment centers on adherence and visual outcomes in patients with vision-threatening diabetic retinopathy (VTDR) in low-resource settings is not well known. This study evaluated adherence to initial laser treatment referral and best-corrected visual acuity (BCVA) at the time of referral, before and after the installation of a regional ophthalmic laser in northwest Tanzania (TZ).

Materials and methods

This was a retrospective cohort study conducted at a single tertiary care institution in northwest TZ. The study included adults with diabetes mellitus (DM) who were diagnosed with VTDR by ophthalmologists at Bugando Medical Centre in Mwanza, TZ, and referred for initial laser treatment between March 1, 2017, and May 1, 2019. Data were collected on patient demographics, VTDR classification (severe non-proliferative diabetic retinopathy, proliferative diabetic retinopathy, and/or clinically significant macular edema), BCVA at the time of referral, and referral adherence. Patients were categorized into two groups based on the location of available laser treatment centers at the time of their referral. The off-site group included patients referred to laser centers located out of the region before treatment became available locally. The on-site group included patients referred for laser treatment within the region after BMC began providing laser therapy for VTDR. Data from the off-site group was compared with data from the on-site group.

Results

A total of 68 patients were included, 32 referred off-site and 36 on-site. Referral for laser treatment on-site was associated with a greater rate of adherence compared to off-site (32/36 (89%) vs 21/32 (66%), *P *= 0.021). Greater adherence to on-site referrals was independent of sex (*P* = 0.024). The proportion of patients with at least moderate vision impairment (BCVA worse than 6/18) in the eye referred for laser was significantly higher among those referred off-site compared to on-site (23/32 (72%) vs 15/36 (42%), *P* = 0.012).

Conclusions

Greater adherence to initial laser treatment referral and better BCVA at the time of referral, providing a better baseline visual acuity to preserve, were observed when laser treatment was available on-site (within region) compared to off-site (out of region). These findings suggest that a systematic approach to improving regional access to laser treatment for adults with VTDR in TZ who must currently travel long distances for care could help delay or prevent blindness due to VTDR. These results have broad implications for other low-resource settings; further study is recommended.

## Introduction

Sub-Saharan Africa (SSA) is projected to experience the largest percentage increase in the incidence of diabetes mellitus (DM) of any region in the world by the year 2045, from 19.4 million (2019) to 47.1 million people (2045) (143%) [1]. Worldwide, all individuals with DM are at risk of developing diabetic retinopathy (DR), such that the global burden of DR is expected to rise in parallel with the increasing prevalence of DM. Data on the prevalence of DR, clinically significant macular edema (CSME), and vision-threatening diabetic retinopathy (VTDR) in SSA is limited by a lack of reporting from all regions in SSA and inconsistent reporting methods. In a systematic review of data from 12 countries in SSA, the reported prevalence range for any DR was 13-82.6% and for VTDR 2.1-51.4% [2]. Worldwide, approximately 35% of people with DM have some degree of DR, and about 10% have VTDR [3]. Based on these proportions, the number of individuals with VTDR in SSA could reach approximately 4.7 million by 2045.

The corresponding increase in DM-associated chronic microvascular complications presents a challenge to healthcare systems. Microvascular changes in the retina associated with DM, termed DR, may cause progressive retinal damage leading to blindness. Typical fundus features of DR include microaneurysms, hard exudates composed of lipid and proteinaceous material, macular edema, and abnormal growth of new blood vessels [4]. Severe proliferation of retinal blood vessels can lead to vitreous hemorrhage or retinal detachment. If macular edema occurs close to the fovea, termed CSME, central vision can be affected. These severe retinal changes are referred to as VTDR [5].

Timely treatment of VTDR with laser photocoagulation can stabilize visual acuity (VA) before irreversible vision impairment or blindness occurs [6,7]. More recently, a synthetic antibody injected into the vitreous of the eye that binds to vascular endothelial growth factor (anti-VEGF) has been approved to treat VTDR. However, due to challenges related to the administration of anti-VEGF agents, including ensuring sterility, the need for repeated injections requiring multiple visits, and the cost of purchase, laser treatment is a mainstay of treatment for VTDR in many areas of SSA [8].

Bugando Medical Centre (BMC) is a referral hospital in the Mwanza region of northwest Tanzania (TZ) serving 13 million people. In a previous study of patients with DM attending outpatient clinics at BMC, 61/91 (67%) individuals were found to have some degree of DR, with 24/91 (26%) diagnosed with VTDR [9]. Due to the high prevalence of VTDR in northwest TZ relative to neighboring regions [10], a 532 nm frequency-doubled Nd-YAG green ophthalmic laser was installed at BMC in April 2018. Prior to this installation, adults diagnosed with VTDR at BMC were referred to laser centers outside the region, typically requiring a 12-hour drive by car.

Adherence to laser treatment referral is necessary to prevent blindness from VTDR, as laser treatment can preserve vision but does not restore vision already lost [11]. Various factors may affect adherence to laser treatment recommendations in northwest TZ. The time and cost burden for patients who need to travel long distances to out-of-region centers for treatment is an obvious barrier to adherence [12,13]. Less obvious barriers include factors affecting patient understanding of recommended treatment. The word “laser” in the Swahili language, *Mionzi*, translates to “radiation” in English. Negative connotations associated with this term may heighten patient anxiety and result in avoidant behaviors that limit adherence [14-17]. Reliance on traditional medicine and healers in northwest TZ may also prevent or delay individuals from consenting to unfamiliar treatment [18].

Currently, fewer than one-fourth of the 25 administrative regions in mainland TZ provide laser treatment within the region (C. Mwanansao, personal communication, January 8, 2024). To better understand the potential benefits of laser treatment available within the region for VTDR in TZ, we aimed to determine if adults with DM followed in clinics at BMC and referred for initial laser treatment had greater adherence to treatment if they were referred to receive laser at BMC compared to referral to a facility outside the Mwanza region. Given laser photocoagulation can stabilize vision but generally does not restore vision already lost due to due to VTDR, an additional aim was to compare BCVA in the eye referred for laser at the time of the initial referral, stratified by referral site, as an indirect measure of long-term vision potential. If greater adherence to laser treatment recommendations and better baseline BCVA at the time of referral are associated with the availability of on-site laser, this may have important public health implications for investing in laser equipment and training ophthalmologists to administer laser photocoagulation in other regions of Tanzania.

## Materials and methods

This retrospective cohort study was conducted at BMC, a tertiary referral hospital in the Mwanza region of northwest TZ. Adults with DM who were evaluated in the BMC ophthalmology clinic between March 1, 2017, and May 1, 2019, and referred for initial laser treatment for VTDR were eligible for inclusion. Informed consent for participating in this study and open access publication was obtained or waived by all participants in this study. The study received initial approval from the Weill Cornell Medicine Institutional Review Board (Protocol No. 1504016121) on May 5, 2017. The most recent continuing review was approved on May 14, 2025.

Inclusion and exclusion criteria 

Inclusion criteria were: (1) age ≥18 years, (2) diagnosis of DM, and (3) referred for initial laser treatment for VTDR during the study period. VTDR was defined as the presence of severe non-proliferative diabetic retinopathy (NPDR), proliferative DR (PDR), and/or CSME with exudates within one disc diameter of the fovea in at least one eye, based on dilated fundus examination [19]. Individuals with PDR-related complications requiring surgical intervention (e.g., retinal detachment) or incomplete data were excluded. 

Data collection

Data were collected for key study variables, including demographic information (age and sex), category of VTDR (NPDR, PDR, and/or CSME), and BCVA in each eye, measured using a standard Snellen chart at a testing distance of 6 meters, at the time of referral for laser treatment. Adherence following referral was recorded as either attended or did not attend a laser clinic appointment. The location of the laser center was noted for individuals referred off-site. Medical records were reviewed, and relevant data were extracted. If referral adherence was not documented, follow-up phone calls were made to patients to determine whether they attended a laser treatment appointment.

Study process

Participants were divided into two groups based on referral location. The off-site group included patients referred to laser centers outside the Mwanza region (i.e., the Kilimanjaro or Dar es Salaam region) before on-site laser treatment became available at BMC (March 2017 to March 2018). The on-site group included patients referred for laser treatment at BMC after laser services became available locally at BMC (April 2018 to May 2019). The cost of laser treatment at a laser center, off-site or on-site, was paid by the TZ national health insurance. However, travel expenses, or time taken off from work to travel to or attend an appointment for laser treatment, were not reimbursed. 

Statistical analysis

BCVA at the time of referral was categorized according to World Health Organization (WHO) reporting criteria: moderate visual impairment was defined as BCVA worse than 6/18 but better than or equal to 6/60, and severe visual impairment as BCVA worse than 6/60 [20]. Data were analyzed using Stata Statistical Software: Release 17 (StataCorp LLC, College Station, Texas, United States). Continuous variables were summarized as mean ± standard deviation (SD), and categorical variables as percentages. Data for the on-site group was compared with the off-site group. Means were compared using an independent samples two-tailed t-test, and proportions were compared using a chi-square test. Logistic regression was performed to evaluate the binary adherence outcome, adjusting for sex. A *P* value < 0.05 was considered statistically significant.

## Results

Approximately 300 adults with DM were seen in the ophthalmology clinic during the study period. A total of 75 patients referred for initial laser treatment were identified. After excluding seven for incomplete data, 68 patients were included in the analysis. Of these 68, 32 (47%) were referred for laser treatment off-site and 36 (53%) were referred on-site. There was no association between age, sex, or category of VTDR and the site referred to (Table 1). A greater proportion of patients referred for treatment on-site attended their laser appointment and received laser treatment compared to those referred off-site (32/36 (89%) vs 21/32 (66%), P = 0.021) (Table 1). Logistic regression of adherence on a dichotomous on-site variable controlling for sex showed patients referred on-site were more than four times as likely to be adherent compared to patients referred off-site (Odds Ratio 4.2, 95% CI 1.21, 16.19; *P* = 0.024). 

**Table 1:** Comparison of baseline characteristics and adherence by referral site ^a ^In eye referred for laser,^ b ^Attended appointment for laser treatment for eye that was referred *: t-score indicates a two-sided independent samples t-test, χ2 indicates chi-square test **: P-values less than 0.05 were considered statistically significant NPDR: non-proliferative diabetic retinopathy; PDR: proliferative diabetic retinopathy; CSME: clinically significant macular edema; BCVA: best-corrected visual acuity

Factor	Referred Off-site (n = 32)	Referred On-site (n = 36)	Test statistic*	P**
Female sex, number (%)	16 (50)	14 (39)	χ2= 0.84	0.36
Age (years), mean ± SD	59.2 (7.0)	60.2 (6.0)	t-test= 0.59	0.56
Reason for referral, number (%)^a^			χ2= 2.84	0.59
PDR	14 (44)	15 (42)		
CSME	14 (44)	11 (31)		
Severe NPDR	2 (6)	4 (11)		
CSME and severe NPDR	1 (3)	3 (8)		
CSME and PDR	1 (3)	3 (8)		
BCVA at time of referral, number eyes (%)				
BCVA worse than 6/18 in eye referred for laser	23 (72)	15 (42)	χ2 = 6.27	0.012
BCVA worse than 6/18, total number eyes	41 (64)	30 (42)	χ2 = 6.81	0.0091
BCVA worse than 3/60, total number eyes	21 (33)	20 (28)	χ2​​​​​​​ = 2.69	0.26
Adherence^b^				
Adherent overall, number (%)	21 (66)	32 (89)	χ2​​​​​​​ = 5.33	0.021
Female sex adherent, number (%)	7 (22)	12 (33)	χ2​​​​​​​ = 0.096	0.76

At the time of referral, a greater proportion of patients referred off-site had at least moderate vision impairment (BCVA worse than 6/18) in the eye referred for laser treatment compared to patients referred on-site (23/32 (72%) vs 15/36 (42%), P = 0.012) (Table 1). A greater proportion of patients referred off-site also had at least moderate vision impairment in one or both eyes compared to patients referred on-site (41/64 (64%) vs 30/72 (42%), P = 0.0091). However, at the time of laser referral, there was no difference in the proportion of patients referred off-site compared to on-site with more severe vision impairment (BCVA worse than 3/60) in one or both eyes (21/64 (33%) vs 20/72 (28%), P = 0.26)).

## Discussion

In this single-center study in the Mwanza region of northwest TZ, nearly all patients with VTDR who were referred for initial laser therapy on-site, at the same hospital where the referral was made, adhered to the treatment recommendation. In contrast, among patients referred to an out-of-region laser center due to local laser treatment being unavailable at that time, only two-thirds were adherent and attended a laser treatment appointment. 

Analysis of potential explanatory variables for this difference in adherence showed no statistically significant difference in sex, age, or category of retinopathy between the on-site and off-site referral groups. These results are similar to those of a study examining patient-related factors and adherence characteristics for diabetic retinopathy screening in a high-resource area, where age, sex, and severity of retinopathy were not associated with positive compliance rates [11]. 

A likely contributor to improved adherence among patients referred for on-site laser treatment is reduced travel time and time lost from work. Patients with VTDR referred on-site avoid the logistical and financial burden of traveling long distances to access care at out-of-region centers. While geographic proximity offers clear advantages, on-site access may also provide intangible benefits that support adherence. Individuals referred on-site in our study were already being followed in the ophthalmology clinic at BMC and would receive laser treatment from familiar providers. Since anxiety has been reported as a reason for missing initial laser appointments [14], familiarity with the treating physician and clinical setting may help reduce anxiety and promote adherence.

Anxiety related to diabetes itself may also impact differentials in treatment adherence. The daily management of diabetes has been associated with a high incidence of clinical and sub-clinical anxiety among people with DM [21]. In a study of adults with type 2 diabetes, individuals with anxious coping styles (i.e., avoidance, escape, or denial) showed reduced adherence to diabetes self-care regimens and poorer glycemic control [22]. Given the considerable effort and resources needed to travel to a laser center out of region, individuals referred off-site for laser treatment and experiencing anxiety due to DM may be more likely to fall back on coping mechanisms such as avoidance, leading to non-adherence.

Our results showed that higher adherence to laser referral on-site is independent of sex is important given documented gender disparities in accessing eye care. Globally, women do not access eye care services as often as men [23]. On a global basis, women are more impacted by vision loss associated with DR compared to men, with the most severe gender imbalance observed in eastern SSA [24]. While our adherence results are not controlled for educational level or economic status, our findings suggest gender is not a barrier to accessing laser treatment services within the region. 

In addition to higher adherence, a greater proportion of patients referred on-site had better VA (better than moderate visual impairment by WHO criteria) in the eye referred for laser treatment compared to those referred off-site. When laser treatment is only available out of region, the awareness of the time and cost required for travel may discourage adults with DM from attending routine appointments for DR screening, leading to lost opportunities to detect and treat VTDR before moderate to severe vision impairment has occurred. Conversely, heightened awareness of local treatment options when a laser is available on-site may encourage patients with DM to be screened in an ophthalmology clinic earlier in the course of the disease, so that BCVA is less compromised at the time of laser referral. Routine screening is especially critical given the painless and progressive nature of DR, which can advance significantly before symptoms of vision loss are noticed.

Earlier self-referral to the ophthalmology clinic for DR screening alone may not fully explain the better BCVA observed among patients referred on-site for laser treatment. A prior survey of patient preferences in DR care found most patients favored a shared decision-making model with their ophthalmologist [25]. For off-site referrals, ophthalmologists at BMC must weigh not only clinical factors but also the substantial logistical burden of travel faced by patients and their families when recommending laser treatment. Patients may choose to delay or decline travel to an out-of-region laser center for a disease that does not cause pain due to the cost, time, and effort required. In contrast, when a laser is available on-site, referral decisions can be based more directly on clinical need. This may facilitate more timely referrals for laser treatment, potentially reducing the risk of further disease progression that compromises VA. 

Overall, our results suggest building laser treatment capacity within the region may have a dual impact to ease the burden of DM-related blindness in TZ. A greater rate of adherence to laser treatment referrals, and less vision impairment at the time of referral, indicating a better VA baseline to preserve, taken together, suggest long-term visual outcomes may be better when patients with VTDR do not have to travel out of region to access laser treatment. This is the first study, to our knowledge, analyzing characteristics associated with utilization of laser treatment resources for VTDR in a low-resource setting. The need for strategies to ease the burden of DM related vision impairment and blindness in TZ is highlighted by the large projected percentage increase in the number of people diagnosed with DM in SSA by 2045 [1]. 

Study strengths include directly comparing utilization of laser resources and baseline VA in the eye referred for laser before and after the introduction of laser access in a low-resource region. Limitations include not demonstrating results are generalizable to other regions in TZ or low-resource settings in other countries, and a lack of data on long-term VA outcomes. However, long-term data on VA in relation to on-site or off-site laser treatment outcomes may have been confounded by the ability of patients initially treated off-site to subsequently receive additional laser treatment on-site after the introduction of laser to BMC. Other limitations include a lack of financial data on costs borne by patients for travel to and from the laser center, and patient socioeconomic status and educational level. While travel costs are not included in the analysis, patients referred off-site would have incurred costs associated with lengthy travel out of the region that were not required for patients referred on-site.

## Conclusions

Patients in northwest TZ with DM and VTDR may have better visual outcomes with continued follow-up and treatment when there is regional access to laser treatment. A systematic approach to providing within-region access to laser treatment for adults with VTDR may be an effective way to delay or prevent blindness due to VTDR in TZ. Future multi-center studies in other low-resource settings may complement our findings and provide guidance for policy decisions. 
